# SARS-CoV-2 Immunization Orchestrates the Amplification of IFNγ-Producing T Cell and NK Cell Persistence

**DOI:** 10.3389/fimmu.2022.798813

**Published:** 2022-02-14

**Authors:** Lucia La Sala, Sara Gandini, Antonino Bruno, Raffaele Allevi, Matteo Gallazzi, Pamela Senesi, Maria Teresa Palano, Paola Meregalli, Ermanno Longhi, Carmen Sommese, Livio Luzi, Emilio Trabucchi

**Affiliations:** ^1^ Lab of Cardiovascular Diabetology and Dysmetabolic Disease, Istituto di Ricovero e Cura a Carattere Scientifico (IRCCS) MultiMedica, Milan, Italy; ^2^ Department of Experimental Oncology, European Institute of Oncology, Istituto di Ricovero e Cura a Carattere Scientifico (IRCCS), Milan, Italy; ^3^ Laboratory of Innate Immunity, Istituto di Ricovero e Cura a Carattere Scientifico (IRCCS) MultiMedica, Milan, Italy; ^4^ Department of Biomedical and Clinical Sciences “L. Sacco”, University of Milan, Milan, Italy; ^5^ Laboratory of Immunology and General Pathology, Department of Biotechnology and Life Sciences, University of Insubria, Varese, Italy; ^6^ Department of Biomedical Sciences for Health, University of Milan, Milan, Italy

**Keywords:** CD4, CD8, NK, immune response, SARS-CoV-2, vaccines

## Abstract

A successful vaccination would represent the most efficient means to control the pandemic of Coronavirus Disease-19 (COVID-19) that led to millions of deaths worldwide. Novel mRNA-based vaccines confer protective immunity against SARS-CoV-2, but whether immunity is immediately effective and how long it will remain in recipients are uncertain. We sought to assess the effectiveness of a two-dose regimen since the boosts are often delayed concerning the recommended intervals.

**Methods:**

A longitudinal cohort of healthcare workers (HCW, *N* = 46; 30.4% men; 69.6% women; mean age 36.05 ± 2.2 years) with no SARS-CoV-2 infection as documented by negative polymerase chain reaction was immunophenotyped in PBMC once a week for 4 weeks from the prime immunization (Pfizer mRNA BNT162b2) and had received 2 doses, to study the kinetic response.

**Results:**

We identified three risk groups to develop SARS-CoV-2 infection IgG^+^-based (late responders, R^-^; early responders, R^+^; pauci responders, PR). In all receipts, amplification of B cells and NK cells, including IL4-producing B cells and IL4-producing CD8^+^ T cells, is early stimulated by the vaccine. After the boost, we observed a growing increase of NK cells but a resistance of T cells, IFNγ-producing CD4^+^T cells, and IFNγ-producing NK cells. Also, hematologic parameters decline until the boost. The positive association of IFNγ-producing NK with IFNγ-producing CD4^+^T cells by the multiple mixed-effect model, adjusted for confounders (p = 0.036) as well as the correlation matrix (r = 0.6, p < 0.01), suggests a relationship between these two subsets of lymphocytes.

**Conclusions:**

These findings introduce several concerns about policy delay in vaccination: based on immunological protection, B cells and the persistent increase of NK cells during 2 doses of the mRNA-based vaccine could provide further immune protection against the virus, while CD8^+^ T cells increased slightly only in the R^+^ and PR groups.

## 1 Introduction

The continued spreading of SARS-CoV-2 and its variants remains a primary emergency in public health worldwide. Several therapeutics are accepted for COVID-19 management (1–3), but no effective therapy to eradicate SARS-CoV-2 infection exists. Vaccination is currently the prevention strategy most accredited to counteract the COVID-19 pandemic (4). The vaccine BNT162b2, carrying the full-length SARS-CoV-2 spike (*S*) gene, received authorization by the US Food and Drug Administration in December 2020, thanks to the clinical trial attesting immunogenicity and efficacy/safety (5–7). Some observations on the role of immunoglobulins G (IgG) and M (IgM) response, regarding the duration and robustness of immunity to SARS-CoV-2, did not provide a close evaluation of the long-lasting immunological memory. Shreds of evidence indicate that circulating immune memory endures for up to 6 months in recovered COVID-19 patients (8), but there is no evidence that recovered people from COVID-19 having antibodies are protected from a second infection. Recent data showed that the recovered COVID-19 patients have displayed, weeks after symptoms appeared, an immune response carried out by response of T cells CD4^+^ and CD8^+^. Thus, vaccination response in healthy individuals should develop the same immune memory as in the recovered patients, but it is unclear whether novel mRNA-based vaccinations could ameliorate symptoms or limit viral shedding. Several reports described the role of the effector-memory exerted by T cells after 1 week from the boost, with circulating CD8^+^ T cells detectable weeks later (9), concluding that the vaccine against SARS-CoV-2 confers long-term immunization, and introduced the concept that CD8^+^ T cells are the key factors conferring protection. However, a dissimilar response was observed by Oberhardt et al., when comparing the CD8^+^ T cell response of vaccine recipients versus COVID-19 convalescents (10). Nowadays, the unexpected and faster viral shedding leads from the epidemic phase to the pandemic phase with the consequent poor vaccine supplies. This scenario forced the governments to reorganize the immunization program, revising the administration intervals of the boost weeks later (reaching 5 weeks) after the 1^ dose, to cover a higher population, despite the recommended interval of 21 days suggested by Pfizer-BioNTech [2 doses (30 *μ*g, 0.3 ml each) administered intramuscularly, 3 weeks apart] (11). These decisions are probably due to recent data showing early reductions in SARS-CoV-2 infection and symptomatic COVID-19 rates, following first vaccine dose administration (12). On the other hand, current evidence indicated the increasing risk of SARS-CoV-2 infection with time due to waning immunity (11). Clinical trials showed that BNT162b2 induces both humoral and cellular-mediated immunity and protects against symptomatic infection (7), but how long the vaccine will remain effective remains to be evaluated. Here, we examined whether the early immunophenotypic changes that occurred in non-COVID subjects (n = 46) receiving mRNA-based vaccination (BNT162b2), during the interval recommended, might confer an advantage in immune protection for justifying a boost delay. Here, we dynamically traced the evolution of the circulating immune cell landscape, following BNT162b2 vaccination, focusing not only on the T cell repertoire but also on NK cells, whose proper activation, given their relevant regulatory role on both innate and adaptive immunity, further impacts on T cell response, including the context of vaccination.

## 2 Materials and Methods

### 2.1 Study Design and Population

The research and procedure (including the informed consent) were approved by the Independent Ethical Committee Board of IRCCS MultiMedica (application number 470.2021, CE-40.2021), Milan. IRCCS MultiMedica healthcare workers (HCWs), as listed for prioritizing an immunization campaign established by the Italian Ministerial directives and AIFA (Agenzia Italiana Farmaco; http://www.salute.gov.it/imgs/C_17_pubblicazioni_2986_12.12.2020), were invited to adhere to the immunization campaign signing the related informed consent for vaccination (*February–June 2021*). The aforementioned participants were enrolled on the CORE study (Comparison of CD4^+^ and CD8^+^ response in responders and non-responders vaccinated with BNT162b2 mRNA (Comirnaty) anti-COVID-19). Additional consents were signed for the collection and storage of biological materials. All participants met with criterion eligibility.

### 2.2 Study Procedures

About 1 h before the mRNA-based vaccine administration, a nasopharyngeal swab for the detection of SARS-CoV-2, a peripheral whole blood venipuncture for immune-phenotyping, and serological and hemocromocytometric assays was performed. At the starting point of the study, all subjects were not infected by the virus, as demonstrated by their negative q-PCR SARS-CoV-2 test. Blood was collected immediately before mRNA-based vaccine (T_0_), at 24 h after the first dose (T_1_), and at days 7 (T_2_), 14 (T_3_), 21 (T_4_), and 28 (T_5_) (**Figure S1A**).

### 2.3 Laboratory Assay

Blood samples were collected after the venipuncture in tubes with EDTA as anticoagulant and processed routinely. The detection of the complete count of hematological parameters was examined in the clinical laboratory of IRCCS MultiMedica using a Sysmex analyzer (XN-9000, Sysmex Corporation, Kobe, Japan). The morphology was examined on whole blood smear by CellaVision DM 96 Digital Cell Morphology System (Sysmex).

#### 2.3.1 Quantitative Determination of Total Immunoglobulins

Serological tests for the detection of immunoglobulins G (IgG) and M (IgM) were performed using the TGS COVID-19 kit, and a chemiluminescent immunoassay (CLIA) was used for the quantitative determination of specific anti-SARS-CoV-2 IgG and IgM on human serum, employing an indirect two-step immunological method based on chemiluminescence using N (nucleocapsid) and S1-RBD (spike) specific antigens (Technogenetisc srl, Milan, Italy). The cutoff values to assess positivity is considered under WHO International Standard 20/136 (2020, WHO Expert Committee on Biological Standardization. WHO/BS/2020.2403) at >24.2 binding antibody unit (BAU)/mL for IgG and ≥1 index for IgM.

#### 2.3.2 Assessment of SARS-CoV-2 Infection

Nasopharyngeal swabs (UTM, Copan, Brescia, Italy) specimens were collected at the timing (**Figure S1A**). All molecular biology procedures were carried out at the molecular biology laboratory of IRCCS MultiMedica. Nucleic acid RNA extraction and purification from nasopharyngeal swabs were performed using an automated liquid handling workstation (Janus G3, PerkinElmer, Waltham, MA, USA) and chemagic 360 nucleic acid extraction (PerkinElmer). The PerkinElmer^®^ SARS-CoV-2 Real-time RT-PCR Assay was used for the qualitative detection of nucleic acid from the SARS-CoV-2 virus. The assay targets at the specific region are nucleocapsid (N) gene and ORF1ab gene. The TaqMan probes for the two amplicons are labeled with FAM and HEX/VIC fluorescent dyes, respectively, to generate a target-specific signal. The assay includes probes for human RNA target (RNaseP) that is used as an RNA internal control to monitor the process from nucleic acid extraction to fluorescence detection labeled with Cy5 fluorescence dye. The specimen results SARS-CoV-2 detected with Ct ≤ 42 for both or one of the target genes.

### 2.4 Stratification Strategy for Identifying Groups of Late Immunological Response (R-)

The HCWs were stratified based on their IgG positive test after day 7 from the first immunization and named responders (R^+^). All subjects that did not develop positivity within the 28 days were considered pauci responders (PR), whereas those with positivity only at day 28 were defined as late responders (R^-^) (**Tables S1, S2**).

### 2.5 Multicolor Flow Cytometry for Immunophenotyping and Cytokine Detection

#### 2.5.1 Peripheral Blood Sample Preparation

Peripheral blood (PB) samples (20–35 ml of whole blood, in EDTA) were collected and immediately used for total mononuclear cell (MC) isolation. Blood samples were diluted with phosphate-buffered saline (PBS) 1:1 (v/v) then subjected to a density gradient stratification with Ficoll Histopaque (Sigma-Aldrich, St. Louis, MO, USA), at 800×g for 20 min. The white ring interface, enriched in peripheral blood mononuclear cells (PBMC), was collected, washed in PBS, and then stored at –80°C in freezing medium (90% FBS, 10% DMSO).

#### 2.5.2 PBMC Stimulation

Mononuclear cells (MNCs) were thawed, counted, and resuspended at 1 × 10^6^ cells/ml in RPMI-1640 medium (Euroclone), supplemented with 10% fetal bovine serum (FBS) (Euroclone), 2 mM l-glutamine (Euroclone), 100 U/ml penicillin, and 100 μg/ml streptomycin (Euroclone). Cells were exposed for 4 h to the cell stimulation cocktail (Tonbo Biosciences, San Diego, CA, USA), containing brefeldin-A, monensin, ionomycin (IONO), and phorbol-myristate acetate (PMA), at 37°C, 5% CO_2_.

#### 2.5.3 Flow Cytometry Analysis

Following cell stimulation, 3 × 10^5^ PBMCs were stained for 30 min, 4°C, in the dark with the following anti-human antibodies for surface antigen detection: CD45-BUV395 (HI30), CD3-BUV737 [SK7 (also known as Leu-4)], CD4-APC (SK3 (also known as Leu3a)), CD8-BV605 (SK1), CD56-BB700 (NCAM16.2), and CD19-BV650 (SJ25C1), all purchased from BD Biosciences (BD, San Jose, CA, USA) according to the manufacturer’s information. For intracellular cytokine detection, cells were fixed and permeabilized using the Cytofix/Cytoperm Fixation and Permeabilization Kit (BD) for 10 min at 4°C. Cells were then washed in 1× Perm/Wash Buffer (BD) and stained for 30 min, 4°C, in the dark, with IFNγ-BV480 (B27) and IL4-PE (MP4-25D2). Following doublet exclusion and morphological FSC-A/SSC-A setting, total leukocytes were gated on CD45^+^ cells. Samples were acquired using a BD FACS Fortessa X20 analyzer, equipped with 5 lasers. Cell subsets were identified as CD45^+^ cells: CD3^+^ cells (total T cells), CD3^+^CD4^+^ cells (CD4^+^T cells), CD3^+^CD8^+^ cells (CD8^+^ T cells), CD3^-^CD19^+^ cell (B cells), and CD3^-^CD56^+^ (total NK cells). Total NK cells were interrogated for CD56 subsets (CD56^bright^, CD56^dim^). Finally, the production of IFNγ and IL4 was interrogated for CD4^+^ and CD8^+^ T cells, B cells, and total NK cells. Flow data were analyzed using the FlowJo v10 software (Tree Star).

#### 2.5.4 Statistical Analysis

Data were reported as mean (standard error) or median and interquartile range (IQR) for continuous variables, and absolute or relative frequencies, as summary measures of categorical variables. The chi-square or Fisher exact test, Wilcoxon rank test, or Kruskal–Wallis rank-sum test was performed to investigate the association of vaccination responses with hematological parameters. Multivariable mixed-effect models for repeated-measure analysis were adopted to analyze changes in time of hematological parameters and investigate the association with vaccination response. The normal distribution of residuals from fully adjusted models was graphically checked, and transformation was adopted if needed to achieve normality. Line graphs were generated to compare responder (R^+^) and non-responder (R^-^) patients in terms of changes over time. Heatmaps were generated calculating Spearman correlation coefficients and by performing a sparse partial least square-differential analysis (sPLS-DA) selecting the most correlated variables by using the first and second component loading vectors. All reported values were two-sided, and p was considered statistically significant. All analyses were carried out using the RStudio (R version 4.0.0) and SAS (version 9.2) software.

## 3 Results

### 3.1 Baseline Characteristics

Forty-six healthcare worker (HCW) subjects (at baseline: N = 46; 30.4% men; 69.6% women; mean age 36.05 ± 2.2 (SD) years; **Table 1** and **Figure S1B**) enrolled in IRCCS MultiMedica (Milan, Italy) as part of the vaccination campaign promoted by the Italian Ministry of Health were subjected to Pfizer BNT162B2 mRNA injection. The rate of the dropout was higher on the day of the boost (as reported in extended data, **Table S1**). All the individuals enrolled in the study are healthcare workers (HCWs). As such, they all received molecular and antigenic tests for SARS-CoV-2 detection as soon as these tests became available (spring of 2020) and regularly during 2020 and 2021; none of the HCWs enrolled in the study tested positive for SARS-CoV-2 before the vaccination. The presence of comorbidities was verified as well in all the HCWs. Results indicated that 1 was in treatment with methotrexate for autoimmunity disease and was excluded from the study; indeed, the latter resulted to be infected by SARS-CoV-2 on the 14th day from 1^ dose, as reported in the supplementary data file (**Table S2**).

**Table 1:** Baseline characteristics.

Variable	(mean±SD)
Age, y	36.05 ± 2.2
Sex	
* woman (N, %)*	32 (69.6%)
* man (N, %)*	14 (30.4%)
WBC (10^3/uL)	7.3 ± 1.7
RBC (10^6/uL)	4.8 ± 0.4
Hgb (g/dL)	14.1 ± 1.3
HCT (%)	42.8 ± 3.4
MCV (fL)	90.3 ± 4.3
MCHC (g/dL)	32.8 ± 0.9
MCH (pg)	29.6 ± 1.5
PLT (10^3/uL)	250.1 ± 51.7
Neutrophils (10^3/uL)	4.3 ± 1.5
Basophils (10^3/uL)	0.04 ± 0.02
Eosinophils (10^3/uL)	0.1 ± 0.1
Lymphocytes (10^3/uL)	2.3 ± 0.6
Monocytes (10^3/uL)	0.5 ± 0.1
*N* reported previous SARS-CoV-2 Infection (2020 pandemic)	3 (EXCLUDED)
*N* undoubted previous SARS-CoV-2 Infection, IgG^+^-based	1
*N* Infection during the vaccination	1

WBC, whole blood cell; RBC, red blood cell; Hgb, hemoglobin; HCT, hematocrit; MCV, mean corpuscular value; MCHC, mean corpuscular hemoglobin concentration; MHC, mean corpuscular hemoglobin; PLT, platelets.

### 3.2 Impact of mRNA-BNT162b2 on the Kinetics of Immune Cell Prevalence

**Figure 1:**
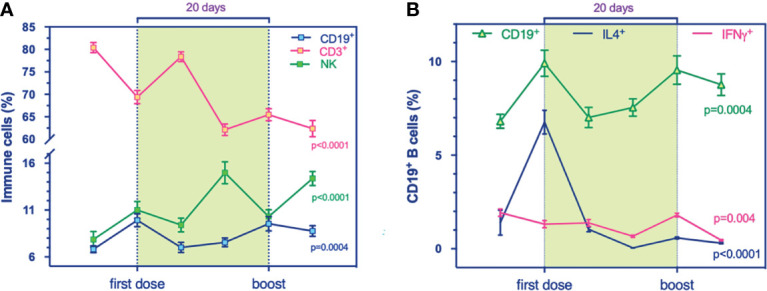
Global landscape of the immune phenotypic changes in a longitudinal cohort who received mRNA-based vaccination against SARS-CoV-2. **(A, B)** Flow cytometry analysis for immune cell compartments panel. **(A)** prevalence % of total T (CD3^+^, purple, p < 0.0001), B (CD19^+^, blue, p = 0.0004), and NK (CD56^+^, green, p < 0.0001) cells during the timing vaccination. **(B)** Prevalence % of IL4^+^-producing B cells (blue, p < 0.0001) and IFNγ^+^-producing B cells (purple, p = 0.004) compared to total B cells (green, p = 0.0004) at the stages of immunization. Multivariable mixed models for repeated measures adjusted for confounders (time, age, sex, responders) were performed.

B cells, T cells, and NK cells are cellular compartments of immunity conferring protection against pathogens. Here we found, during the timing of vaccination, that B cells slightly grew up in number and NK cell frequencies were higher. T cells showed a slow but inexorable decline over time (**Figure 1A**). In addition, multivariable models showed i) a positive association between T cells and IgG levels (p = 0.016, **Table S3**) and ii) a negative association between T cells and NK cells at all times (p < 0.0001, **Table S4**).

### 3.3 Impact of mRNA-BNT162b2 on Early T_H_1/T_H_2 Response

**Figure 2:**
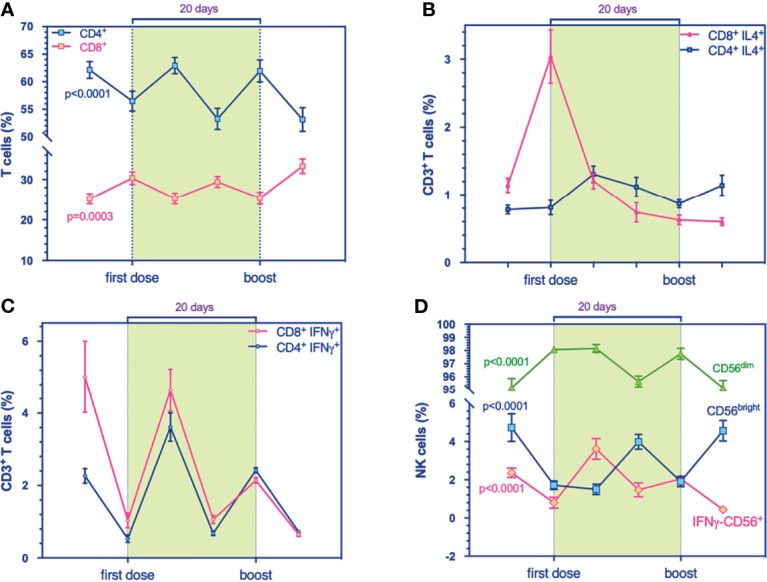
Flow cytometry analysis for immune cell compartments panel. **(A)** Prevalence % of CD4^+^ (blue, p < 0.0001) and CD8^+^ T cells (purple, p = 0.0003). **(B)** Prevalence % of IL4^+^-producing-CD4^+^ (blue) and -CD8^+^ T cells (purple), respectively. **(C)** Prevalence % of IFNγ^+^-producing-CD4^+^ (blue) and -CD8^+^ T cells (purple), respectively. **(D)** Prevalence % of IFNγ^+^-producing NK cells (purple, p < 0.0001), CD56^dim^ (green, p < 0.0001), and CD56^bright^ (blue, p < 0.0001) during the timing. Multivariable mixed models for repeated measures adjusted for confounders (time, age, sex, responders) were performed.

We examined the immunophenotypic changes to evaluate the potential role of mRNA vaccine against SARS-CoV-2 on B cell frequency, as the latter can induce and regulate T cell immune response and can differentiate into effector subsets that secrete polarizing cytokines. Our results revealed a transitory higher production of the B cell compartment the day after both administrations, 1^ dose and the boost, significantly (**Figure 1A**). Also, multicolor flow cytometry was used for assessing longitudinal IL4-producing B cell frequency in PBMC samples (**Figure 1B**). We found a higher percentage of IL4-producing B cells at T_1_ (**Figure 1B**) that decreased over time, suggesting a transitory effect of the vaccine, supported presumably by the amplification trend of T cells (**Figure 2A**). Indeed, the increase of B cell response could be supported by the balancing between T_H_1 and T_H_2 cells, as demonstrated by a recent study on influenza immunization versus those who failed to produce protective antibodies after vaccination (13). The balance of cytokines produced by T_H_1 and T_H_2 subsets is a key factor influencing the character of the immune response. In multivariable models, we showed a positive association between IL4-producing B cells at the boost with IgG levels. This synergic influence of IL4-producing CD8^+^ T cells, increasing within timing (**Figure 2B**), might drive to a humoral immune response, following immunization. Within the stratification groups (R^+^, R^-^, or PR; see classification strategy in the extended data file, **Table S1**), we showed that IL4-producing CD8^+^ T cells overlapped in the groups reaching a peak at T_1_ and then decay, suggesting a transient humoral immunity (**Figure S3A**).

### 3.4 Resistance of IFNγ-Producing Cells During Immunization

IFNγ has an important role in the innate response against intracellular infections and in the regulation of adaptive immune response. Recently, it has been demonstrated that also B cells could produce IFNγ, which normally is mainly produced by NK cells and CD4^+^ T cells for innate strengthening. IFNγ directly inhibits viral replication, protecting the host against virus-induced pathogenesis and lethality (14).

Notably, here we noticed a reduction of IFNγ-producing CD19^+^ (**Figure 1B**), under the increased frequencies of IL4-producing CD8^+^ T cells (**Figure 2B**). Increasing evidence from clinical observations reveals the great heterogeneity of B cells. Among groups, IFNγ-producing B cells showed an increase in R^+^ during the first stages of the primary response (**Figure S3B**) rather than the R^-^ group in early cytotoxic cytokine-producing cells. At 24 h from the first immunization, all groups produced IL-4-expressing B cells (**Figure S3C**) and have increased IFNγ-producing B cells (**Figure S3B**).

The comparison highlighted the prevalence of T_H_1-like response, as showed by IFNγ-producing CD8^+^ (**Figure S3D**) and IFNγ-producing CD4^+^ T cells (**Figure S4A**) within the groups (R^+^, R^-^, and PR).

In addition, we observed at the early stage of immunization a significant expansion of NK cells persisting during the timing (**Figure 1A** and **Figure S6**) and the mixed model for repeated measurements showed statistically significant inverse associations between NK cells and T cells (**Table S4**), adjusted for confounders. In addition, a positive association between IFNγ-NK cells with IFNγ-producing CD4^+^ T cells might explain the synergy of IFNγ production at the first stages of immunization (**Table S5**).

Also, IFNγ may control T cell homeostasis in the context of CD8^+^ T cell memory and protective immunity, a relevant immunological feature, particularly in the context of vaccination. Our results are coherent with the observations of SARS-CoV-2 infection in which NK cells show a decreased cytotoxicity, mediated by IFNγ-NK cells, together with impaired capabilities to produce perforin, granzymes, and IFNγ (15–17).

### 3.5 Delay of IgG Production Associated With Hematological Manifestations During the Whole Immunization and With T Cell Decline

Serum IgG detection is insufficient for the diagnosis of infection or immunization, especially in the first weeks. In this work, we observed an inverse association between IgG levels and neutrophils which becomes stronger over time, and with lymphocytes (positive association) (**Table S6**). Also, B cells in immunophenotyping (**Table S7**) showed associations with IgG growth (see also correlation matrix, **Figure S7**).

Moreover, we also tested the changes in hematologic parameters during the vaccination, with the idea that a mild trend would be observed “vaccine spike-induced,” as COVID-19 symptomatic patients which present severe leukopenia lymphopenia (18), and morphologic alteration in peripheral smears (such as the appearance of prominent toxic and apoptotic changes in granulocytes, mono-lobated neutrophils, leukoerythroblastosis, shift to metamyelocyte stage, and rare blasts) (19).

**Figure 3:**
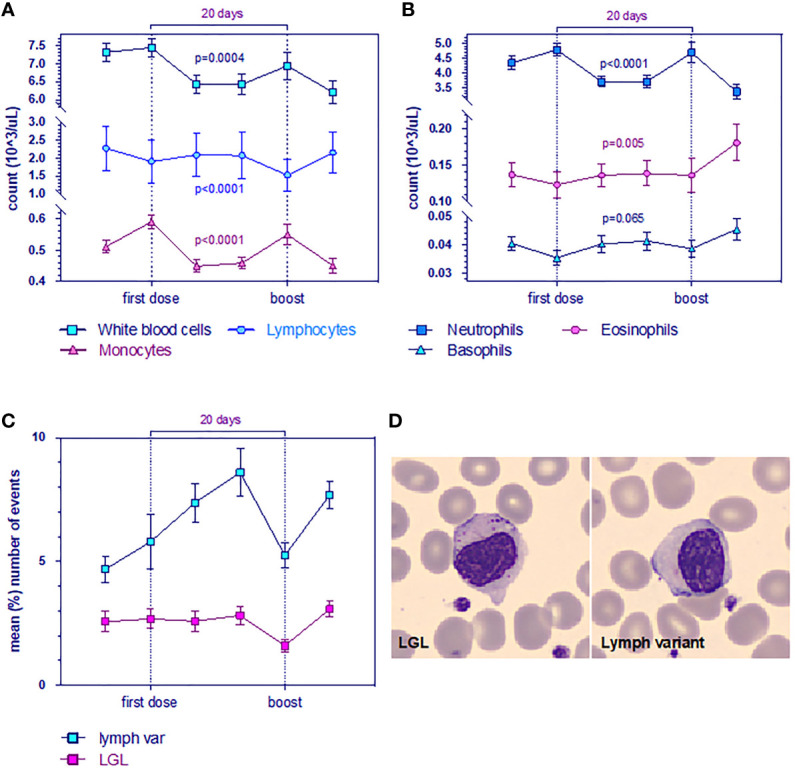
Evolution of leukocyte levels **(A)** White blood cell (WBC, blue), lymphocyte (light blue) and monocyte counts are significantly reduced. Data are mean ± s.e.m. p-value for time from multivariable mixed-effect model for repeated measures. **(B)** Neutrophils (blue), eosinophils (purple), and basophils (light blue). Data are mean ± s.e.m. p-value for time from multivariable mixed-effect model for repeated measures. **(C)** Mean (%) number of events (n = 23 LGL, n = 34 lymphocyte variants) for large granular lymphocytes (LGL, purple) and for the variant form of lymphocytes. Wilcoxon test for pairing showed r = 0.8 (Spearman) and p = 0.03. **(D)** Morphological changes in whole blood smearing stand out low quantities of anomalous cells observed with CellaVision (Sysmex).

In this study, although overall mean counts were in the normal range [as reported even by others (20, 21)], we observed a reduction of total white blood cells (WBC) and mean absolute lymphocyte counts after the 1^ dose, persisting until day 28 significantly over time. Therefore, the number of monocytes and neutrophils, which are increased the day after the 1^ dose, undergoes decay in a significant manner (**Figure 3A**). The inverse trend was observed for the number of eosinophils and basophils, which showed a significant tendency to increase over time (**Figure 3B**).

Peripheral blood smear showed the presence of growing frequencies of atypical lymphocytes (lymphocyte variants) which are well described in the setting of viral infections. We observed that plasmacytoid forms increased over time, whereas no difference was found on large granular lymphocytes (LGL) (**Figures 3C, D**), although a decay after the boost was evident (validated by two board-certified hematopathologists PM and FR).

In COVID-19 patients, lymphopenia could be a prognostic predictor for disease as CD4^+^ T cells, CD8^+^ T cells, B cells, and NK cells decreased, with a significant reduction of CD8^+^ (22).

## 4 Discussion

During COVID-19, T and B cell responses have been tracked in blood samples allowing identification of SARS-CoV-2-specific T_H_1 CD4^+^ and CD8^+^ T cell responses and the presence of SARS-CoV-2-specific neutralizing antibodies in COVID-19 patients (23) and vaccine recipients (6). Apart from T cells, NK cells have been also monitored in SARS-CoV-2 infection, where they have been found to be impaired in their cytotoxic activities, with reduced capability to release IFNγ, perforin, and granzymes. The recent clinical trial demonstrated the efficacy of Comirnaty starting at 28 days in producing antibodies against SARS (7). Starting from these pieces of evidence, in particular the early decrease in COVID-19 rate, the policy delaying of the second boost has become normal practice in countries where vaccine supplies are scarce or where people’s hesitancy predominates. Moreover, the different immune response to the vaccine is the major source of worry in the general population, which is characterized by different ethnic groups, with genetic susceptibility to the risk of infection and the severity of disease symptoms (24).

Here, we sought to establish a) whether the 1^ dose could provide a first evaluation on the production of effector immune cells, exploring the frequencies of cytokine-producing immune cells in a longitudinal cohort of HCW that could represent the general population, and b) the presence of groups with different immune responses, based on IgG positivity.

Our results highlighted the influence of B cells on host immunity at 24 h after the 1^ dose by the prevalence of IL4-producing B cells (typically present after infection and immunization), whose decay over time is preceded by a significant increase at T_1_ (**Figure 1B**). In addition, we observed an increase of IL4-producing CD8^+^ T cells at 24 h after the 1^ dose, suggesting a selective regulation of T_H_2 response in producing antibodies. In particular, we found significant associations with B cells and effector B cells with the IgG production at the second boost. Thus, IL4-producing B cells presumably shift T cells to T_H_2 responses toward B cell production. Consistent with the associations with serum IgG data, the generation of IL4-producing B cells could be critical in driving the susceptibility of T_H_2 immune response to vaccine and enhance IgG production at the boost.

In line with the recent evidence on the regulation of CD4^+^ T helper (Th) cell responses—and the polarization of T helper 1 (T_H_1) and T helper 2 (T_H_2) subsets by producing cytokines like interleukin 4 (IL4) and interferon-gamma (IFNγ), mediated by B cells in independent-antibody function (25, 26)—we observed, during the phase interposed by the first and second doses (T_2_–T_5_), that the cellular subset ratio gets diverted in IFNγ-producing CD8^+^ and -CD4^+^ T cells at T_2_ (**Figure 2C**), which persisted in T_3_ and T_4_. One week after the boost at T_5_, IL4-producing CD4^+^ T cells are the most prominent subset, concomitantly with IFNγ-producing CD4^+^ and -CD8^+^, suggesting an equilibrium in balancing of the subpopulations. Collectively, the character of immune response may be influenced by T_H_1 and T_H_2, and the production of B cells might be influenced by IL4-producing CD8^+^ T cells which might be the key suppressor of IFNγ production.

Also, immunological studies demonstrated the differentiation of naïve B cells into effector B cells by a combination of T cell-dependent signals and the cytokine environment, which finally could influence the outcome of infectious disease. Indeed, we found that B cells increase in frequency (%) during the initial stage of immunization (day 1) while T cell subsets increase during the successive stages (days 7–14). In particular, IFNγ-producing CD4^+^ and IFNγ-producing CD8^+^ T cells are in line with these pieces of evidence, since T-cells as a major source of IFNγ in the adaptive immune response take days to develop a prominent IFNγ response (27).

A relevant issue addressed by our study consisted in the dynamic tracing of NK cells, following vaccination, among the classical T cell repertoire. The frequencies of total NK cells increase during the timing (**Figure S4C**). Generally, IFNγ-producing NK cells decreased, then underwent to increase at T_2_ but then decayed over time (**Figure S4D**). The kinetics of CD56^bright^ overlap (**Figure 2D**) to IFNγ-producing NK cells in T_1_ and T_4_, corresponding to the 1^ dose and the boost, suggesting an effect dependent on the first immunization.

The innate function of B cells has recently attracted considerable attention, as NK and B cells could interact productively. NK cells have been demonstrated to rapidly respond to the acute phase of infection of different viruses, as well as the following vaccination with live-attenuated yellow fever (28–31). Of notice, NK cells are involved in the elimination of virus-infected cells by shaping the adaptive response mediated by T cells (32, 33). Activated NKs are a relevant source of IFNγ, a crucial cytokine that directly exerts cytotoxic effects and indirectly activates second effector arms of both innate and adaptive immunity. IFNγ is largely known to have a crucial role in instructing CD8^+^ T cell expansion and contraction, as adaptive immune responses to several pathogens, including viruses (34, 35).

Indeed, we think that 2 doses of mRNA-based vaccine could supply the NK hypo-responsiveness noted in the pathogenesis of severe SARS-CoV-2 infection (together with the hyperfunction of CD4+ and CD8+ T cells) and might provide further immune protection against the virus. The 2 doses regimen for an effective immune response seems to be necessary.

We next explored the changes affected by hematological parameters. Their reduction, such as the absolute count of lymphocytes, monocytes, and neutrophils, is associated with serological IgG by a multivariable mixed-effect model for repeated measures. Therefore, the variation in the frequency of the atypical form of lymphocytes tends to increase over time and correlates with large granular lymphocytes (LGL vs. variant form of lymphocytes, p = 0.03) (**Figures 3C, D**), which is considered by some authors as NK cells (36).

In conclusion, the requiring of a third dose, or of the enhancement with doses of different vaccines, mRNA, or adenovirus vector, is needed (37). Our vaccinated subjects, whose antibodies are not detected (R^-^), have impaired recruiting of T cells which might explain the reason for the CD8^+^ increase in R^+^ and PR, rather than R^-^, but IFNγ is reduced reflecting the imbalanced T_H_1 and T_H_2 (highlighted in **Figures S5A**–**D**). CD4^+^ is reduced in all groups, and IFNγ undergoes the first increase at T_1_ in R^+^ and then decays; therefore, the protective power conferred on the ability of these cells to produce IFNγ is reduced over time. In the R^+^ group, the % IFNγ-producing B cells increase in the first stage, then decay, then rise to the boost (in the others, they are delayed only at T_4_). The T_H_2 response appears to be prevalent in the groups and helps to intensify the production of antibodies.

Therefore, repetitive boosts should be considered essential in light of these events to intensify the immune response.

## Data Availability Statement

The raw data supporting the conclusions of this article will be made available by the authors, without undue reservation.

## Ethics Statement

The study was conducted according to the guidelines of the Declaration of Helsinki and approved by the Independent Ethical Committee Board of IRCCS MultiMedica (application number 470.2021, CE-40.2021), Milan. The patients/participants provided their written informed consent to participate in this study.

## Author Contributions

The study was designed and planned by LSL and ET and was conducted by LSL, AB, SG, RA, MG, PS, MTP, PM, EL, CS, and LL. SG contributed to the statistical analysis of the data. LSL and ET drafted the manuscript. LSL, AB, and MG were responsible for the acquisition and analysis of cytofluorimetric assay. LSL, RA, PS, and MTP prepared the samples. LSL, PM, and EL interpreted the hematological data. CS and LL revised the manuscript for intellectual content. All authors contributed to the interpretation of the study results, approved the submitted manuscript, and agreed to the integrity of the work. LSL is the guarantor and accepted full responsibility for the work and conduct of the study, had access to the data, and controlled the decision to publish.

## Funding

This study is funded by IRCCS MultiMedica Italian Ministry of Health (Ricerca Corrente) with in-kind support from the “Fondazione Romeo ed Enrica Invernizzi” Milan, Italy. AB has received funds from the Italian Association for Cancer Research (AIRC-MFAG id 22818) and the Cariplo Foundation (id- 2019-1609). The funder did not have any role in the design and conduct of the study; collection, management, analysis, and interpretation of the data; preparation, review, or approval of the manuscript; and decision to submit the manuscript for publication. All authors had full access to all data analysis outputs (reports and tables) and take responsibility for their integrity and accuracy.

## Conflict of Interest

The authors declare that the research was conducted in the absence of any commercial or financial relationships that could be construed as a potential conflict of interest.

The reviewers MC and EC have declared a shared affiliation with the authors RA, PS, and LL to the editor at the time of review.

## Publisher’s Note

All claims expressed in this article are solely those of the authors and do not necessarily represent those of their affiliated organizations, or those of the publisher, the editors and the reviewers. Any product that may be evaluated in this article, or claim that may be made by its manufacturer, is not guaranteed or endorsed by the publisher.
