# Supplementary material for: SARS-CoV-2 Immunization Orchestrates the Amplification of IFNγ-Producing T Cell and NK Cell Persistence
Source: Front Immunol. 2022 Feb 14;13:798813. doi: 10.3389/fimmu.2022.798813 (PMC8882867; doi:10.3389/fimmu.2022.798813)
Supplement: Supplementary file 1 [file DataSheet_1.docx]

Supplementary Material

# Supplementary Data

Manuscript: SARS-CoV2 immunization orchestrates the amplification of IFNγ-producing T cells and NK cells persistance

## *Study design and Demographic characteristics*

## Blood was collected from 46 volunteers’ participants with presumable no symptoms of COVID-19 and subjected to peripheral whole blood venipuncture once a week for four weeks. Six time-points were pointed out. The drop-out of the study at 7 days from the first vaccine injection was 19%, at 14 days was 17%, at 21 days was 37% and at 28 days was 28% (supplemental Table S1). The rate of drop out concomitantly to second dose of vaccine (at day 21) was due to appearance of symptoms such as fever (38° of temperature). In our study, 1 person of HCWs received a positive nasopharyngeal molecular test (qPCR-based) at day 14 after the immunization although the higher IgG levels, and were excluded from the study (Supplemental Fig. S1, S2).

## *Subjects stratification IgG-based response*

## At pre-vaccination (Supplemental Fig. S1), 43 naïve SARS-CoV-2 infections showed undetectable levels of IgG and IgM antibodies specific for N and S1-RBD. The stratification of subjects was assessed by the primary immune response IgG and IgM values. Early humoral immunity mediated by IgG production is induced by vaccine after 7 days from the first injection in 23% of participants (early responders, R^+^). 72% has responded after 14 days from the first dose, 62% has responded after 21 days from the first dose, 87% after 28 days from the first dose (late-responders, R^-^). We identified a group of participants that has early developed a higher IgG levels: at baseline, 8% of participants showed an IgG^+^ levels that were not increased after the first dose (T_1_, 24h), but increased at 7 days, significantly (supplemental Fig. S2, a-c; supplemental Table S2). It was presumable that these levels were due to previous, undetected, exposure to virus but at baseline the molecular test for CoV-2 infection was negative. Only in 3 participants reported a mild COVID-19 history (Table S1, S2). In these subjects (early responders, R^+^) the frequency of IgG^+^ was increased during the first and second doses.

## Supplemental Tables

## Table S1. Seroconversion rates during the timing and drop out

| TIME-POINTS | SEROLOGY RESULTS | | N HCWs drop-out (%) |
| --- | --- | --- | --- |
|  | **N IgG positive (%)** | **N IgM positive (%)** |  |
| T_0_ (1-2 h ante) | 3 (8) * | 1 (2.5) * | 4 (9) |
| T_1_ (d 1) | 3 (8) | 1 (2.5) | 6 (13) |
| T_2_ (d 7) | 9 (23) | 7 (18) | 9 (19) |
| T_3_ (d 14) | 28 (72) | 12 (31) | 8 (17) |
| T_4_ (d 21) | 23 (62) | 5 (13) | 17 (37) |
| T_5_ (d 28) | 32 (87) | 16 (43.5) | 13 (28) |
|  |  |  |  |
| Groups | **N (%)** | **N (%) with IgG^+^ (BAU/mL) at T_0_** |  |
| R- | 11 (28) | 0 (0) |  |
| PR | 19 (49) | 0 (0) |  |
| R+ | 9 (23) | 4 (10) |  |

## T_0_: 1-2 hours before the injection of first immunization with mRNA-based BNT162b2, Pfizer/BioNtech; T_1_: 24 hours post-first immunization; T_2_: day 7 post-immunization; T_3_: day 14 post-immunization; T_4_: day 1 post-boost (day 21 post-1^immunization); T_5_: day 7 post-boost (day 28 post-1^immunization). * previous mild symptoms, but the infection was not confuted by molecular test. The stratification of the subjects was based on IgG serological results: 17.9 – 24.2 doubt; <17.9 negative; >24.2 positive. R- (late-responders), PR (pauci responders), R+ (responders).

## Table S2. CLIA Ab anti-N and anti-S1 RBD IgG (BAU/mL) and IgM (Index)

## In grey, subjects who has a mild history of COVID-19 in 2020. The ID=5 was excluded from the study. In yellow (ID=24), a participant who positivized at day 14 with mild symptoms as diarrhea, headache, and a history of autoimmunity disease. Negative SARS-Cov-2 detection in all time-points was assessed.

##
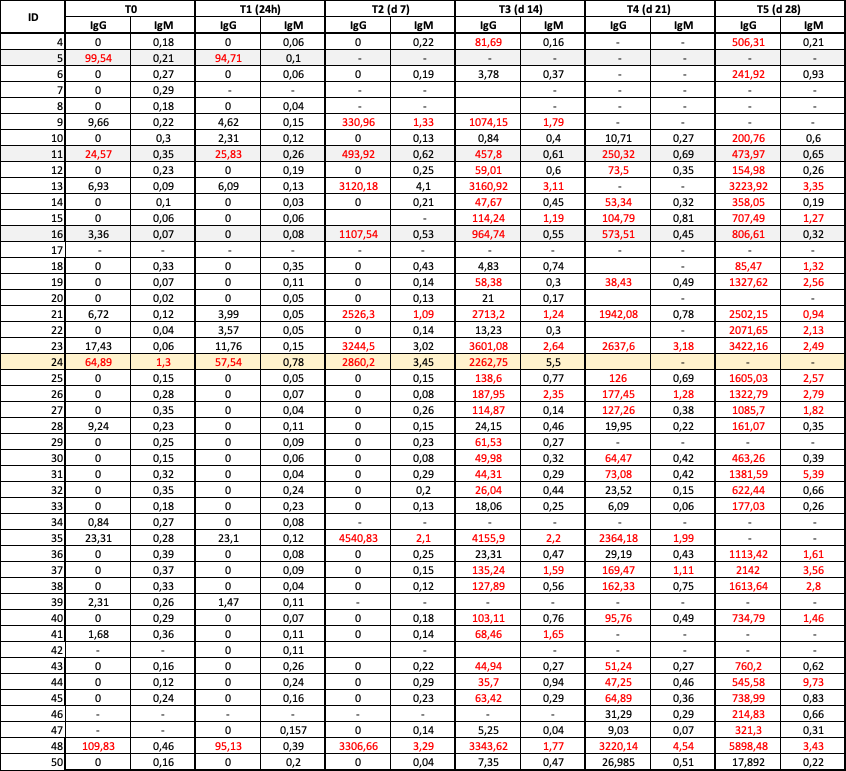


## Table S3. Associations of Immunoglobulins IgG response with CD3^+^ T cell over time

| Effect | Response |  |  |  |
| --- | --- | --- | --- | --- |
|  |  | **IgG T2** | | |
|  |  | **Estimat.** | **SE** | **P-value** |
| Intercept |  | 553.27 | 111.48 | <.0001 |
| baseline |  | 22.47 | 7.64 | 0.0074 |
| Age |  | -4.94 | 1.91 | 0.0164 |
| Sex | **Female** | -181.26 | 62.40 | 0.008 |
|  | **Male** | 0 | . | . |
| Response | **PAUCI-R** | 26.32 | 67.95 | 0.7021 |
|  | **R+** | 472.64 | 105.86 | 0.0002 |
|  | **R-** | 0 | . | . |
| Time | **1** | -526.91 | 85.78 | <.0001 |
|  | **2** | -289.21 | 85.78 | 0.0011 |
|  | **3** | -265.19 | 85.78 | 0.0026 |
|  | **4** | -314.12 | 92.19 | 0.0009 |
|  | **5** | 0 | . |  |
| CD3^+^ T cell |  | 8.28 | 3.20 | **0.0166** |

## Multivariable mixed effects modelling revealed associations among IgG and the increase of CD3^+^ T cells over time, adjusted for confounders. Estimation for different times. SE: standard error.

## Table S4. Associations of the response NK cells with T cells over time

## Multivariable mixed effects modelling revealed associations among total NK cells and the increase of CD3^+^ T cells over time, adjusted for confounders. Estimation for different times. SE: standard error.

| Effect | Response |  |  |  |  |  |  |  |  |  |
| --- | --- | --- | --- | --- | --- | --- | --- | --- | --- | --- |
|  |  | **NK T1** | | | **NK T2** | | | **NK T3** | | |
|  |  | **Estimat.** | **SE** | **P-value** | **Estimat.** | **SE** | **P-value** | **Estimat.** | **SE** | **P-value** |
| Intercept |  | 35.20 | 3.98 | <.0001 | 32.79 | 5.58 | <.0001 | 25.48 | 3.44 | <.0001 |
| baseline |  | -0.02 | 0.03 | 0.50 | 0.03 | 0.03 | 0.27 | 0.03 | 0.03 | 0.25 |
| Age |  | -2.20 | 0.76 | 0.01 | -1.03 | 0.92 | 0.27 | -2.05 | 0.80 | 0.02 |
| Sex | **Female** | 0.00 | . | . | 0.00 | . | . | 0.00 | . | . |
|  | **Male** | -1.69 | 0.91 | 0.07 | 0.22 | 0.91 | 0.81 | 0.12 | -0.28 | 0.92 |
| Response | **PAUCI-R** | -4.17 | 1.12 | 0.00 | -2.17 | 1.06 | 0.05 | 0.83 | -1.93 | 1.13 |
|  | **R+** |  | 0.00 | . | 0.00 | . | . | 0.00 | 0.00 | . |
|  | **R-** | -6.95 | 1.29 | <.0001 | -6.15 | 1.29 | <.0001 | -6.07 | 1.30 | <.0001 |
| Time | **1** | -3.68 | 1.28 | 0.00 | -4.27 | 1.32 | 0.00 | -3.42 | 1.33 | 0.01 |
|  | **2** | -5.48 | 1.31 | <.0001 | -5.15 | 1.28 | <.0001 | -5.11 | 1.32 | 0.0002 |
|  | **3** | 0.64 | 1.29 | 0.62 | 0.15 | 1.29 | 0.91 | 0.54 | 1.29 | 0.68 |
|  | **4** | -4.47 | 1.37 | 0.001 | -4.08 | 1.43 | 0.01 | -4.01 | 1.42 | 0.01 |
|  | **5** | 0.00 | . | . | 0.00 | . | . | 0.00 | . | . |
| CD3^+^ T cells |  | -0.24 | 0.05 | <.0001 | -0.23 | 0.07 | <0.001 | -0.16 | 0.05 | <0.001 |

| Effect | Response |  |  |  |  |  |  |
| --- | --- | --- | --- | --- | --- | --- | --- |
|  |  | **NK T4** | | | **NK T5** | | |
|  |  | **Estimat.** | **SE** | **P-value** | **Estimat.** | **SE** | **P-value** |
| Intercept |  | 23.64 | 4.31 | <.0001 | 19.42 | 2.69 | <.0001 |
| baseline |  | 0.07 | 0.03 | 0.05 | 0.05 | 0.03 | 0.04 |
| Age |  | -0.71 | 0.91 | 0.45 | -1.30 | 0.80 | 0.12 |
| Sex | **Female** | 0.00 | . | . | 0.00 | . | . |
|  | **Male** | 0.12 | 0.11 | 1.05 | 0.12 | 1.22 | 0.91 |
| Response | **PAUCI-R** | 0.83 | -2.73 | 1.32 | 0.83 | -0.60 | 1.20 |
|  | **R+** | 0.00 | 0.00 | . | 0.00 | 0.00 | . |
|  | **R-** | -6.76 | 1.43 | <.0001 | -6.40 | 1.27 | <.0001 |
| Time | **1** | -3.30 | 1.43 | 0.02 | -3.21 | 1.29 | 0.01 |
|  | **2** | -5.00 | 1.47 | 0.001 | -4.71 | 1.29 | 0.0004 |
|  | **3** | 0.56 | 1.43 | 0.70 | 0.12 | 1.26 | 0.92 |
|  | **4** | -4.44 | 1.41 | 0.002 | -4.02 | 1.32 | 0.003 |
|  | **5** | 0.00 | . | . | 0.00 | . | . |
| CD3^+^ T cells |  | -0.15 | 0.07 | 0.03 | -0.10 | 0.04 | 0.01 |

**Table S5. Associations of the response IFNγ-producing NK cells with IFNγ-producing CD4+ T cells over time**

Multivariable mixed effects modelling revealed associations among IFNγ-producing NK cells and the increase of IFNγ-producing CD4+ T cells over time, adjusted for confounders. Estimation for different times. SE: standard error.

| Effect | Response |  |  |  |  |  |  |  |  |  |
| --- | --- | --- | --- | --- | --- | --- | --- | --- | --- | --- |
|  |  | **IFNγ-producing NK T1** | | | **IFNγ-producing NK T2** | | | **IFNγ-producing NK T3** | | |
|  |  | **Estimat.** | **SE** | **P-value** | **Estimat.** | **SE** | **P-value** | **Estimat.** | **SE** | **P-value** |
| Intercept |  | 0.416 | 0.648 | 0.526 | 0.062 | 0.737 | 0.933 | -0.465 | 0.784 | 0.558 |
| baseline |  | -0.009 | 0.010 | 0.408 | -0.006 | 0.011 | 0.556 | -0.016 | 0.010 | 0.109 |
| Age |  | 0.096 | 0.323 | 0.768 | -0.317 | 0.339 | 0.357 | 0.414 | 0.354 | 0.251 |
| Sex | **Female** | 0.000 | . | . | 0.000 | . | . | 0.000 | . | . |
|  | **Male** | -0.384 | 0.374 | 0.314 | -0.041 | 0.395 | 0.919 | 0.121 | 0.397 | 0.764 |
| Response | **PAUCI-R** | 1.104 | 0.471 | **0.026** | 0.515 | 0.499 | 0.310 | 0.826 | 0.440 | 0.070 |
|  | **R+** | . | 0.000 | . |  | 0.000 | . | 0.000 | . | . |
|  | **R-** | 2.158 | 0.560 | **0.000** | 2.119 | 0.566 | **0.000** | 2.165 | 0.531 | **<.0001** |
| Time | **1** | 0.360 | 0.518 | 0.488 | 0.310 | 0.535 | 0.563 | 0.506 | 0.497 | 0.311 |
|  | **2** | 3.215 | 0.530 | **<.0001** | 3.130 | 0.517 | **<.0001** | 3.241 | 0.494 | **<.0001** |
|  | **3** | 0.966 | 0.522 | 0.066 | 1.145 | 0.520 | **0.029** | 1.054 | 0.484 | 0.031 |
|  | **4** | 1.617 | 0.555 | **0.004** | 1.573 | 0.579 | **0.008** | 1.664 | 0.530 | **0.002** |
|  | **5** | 0.000 | . | . | 0.000 | . |  | 0.000 | . | . |
| IFNγ-producing CD4^+^ T cells |  | 0.558 | 0.253 | **0.036** | 0.207 | 0.094 | **0.035** | 1.430 | 0.543 | **0.013** |

| Effect | Response |  |  |  |  |  |  |
| --- | --- | --- | --- | --- | --- | --- | --- |
|  |  | **IFNγ-producing NK T4** | | | **IFNγ-producing NK T5** | | |
|  |  | **Estimat.** | **SE** | **P-value** | **Estimat.** | **SE** | **P-value** |
| Intercept |  | -1.018 | 1.460 | 0.493 | 1.527 | 0.813 | 0.072 |
| baseline |  | -0.005 | 0.014 | 0.698 | -0.012 | 0.010 | 0.214 |
| Age |  | -0.200 | 0.407 | 0.629 | -0.040 | 0.309 | 0.899 |
| Sex | **Female** | 0.000 | . | . | 0.000 | . | . |
|  | **Male** | 0.121 | -0.345 | 0.450 | 0.121 | -0.526 | 0.337 |
| Response | **PAUCI-R** | 0.826 | 1.470 | 0.571 | 0.826 | 1.082 | 0.452 |
|  | **R+** | 0.000 | 0.000 | . | 0.000 | 0.000 | . |
|  | **R-** | 2.242 | 0.651 | **0.001** | 2.370 | 0.512 | **<.0001** |
| Time | **1** | 0.350 | 0.605 | 0.564 | 0.598 | 0.468 | 0.203 |
|  | **2** | 3.507 | 0.625 | **<.0001** | 2.934 | 0.467 | **<.0001** |
|  | **3** | 0.986 | 0.605 | 0.106 | 1.239 | 0.454 | **0.007** |
|  | **4** | 1.552 | 0.599 | **0.011** | 1.678 | 0.478 | **0.001** |
|  | **5** | 5.000 | 0.000 | . | 0.000 | . | . |
| IFNγ-producing CD4^+^ T cells |  | 0.723 | 0.500 | 0.163 | -0.763 | 0.681 | 0.273 |

## Table S6. Associations of neutrophil and lymphocytes counts (10^3/uL) with IgG response over time

## Multivariable mixed effects modelling revealed associations among IgG and the increase of B cells over time, adjusted for confounders.

## Estimation for different times. SE: standard error.

| Effect | Response |  |  |  |  |  |  |
| --- | --- | --- | --- | --- | --- | --- | --- |
|  |  | **Neutrophils** | | | **Lymphocytes** | | |
|  |  | **Estimat.** | **SE** | **P-value** | **Estimat.** | **SE** | **P-value** |
| Intercept |  | 36.08 | 4.5 | **<.0001** | 0.65 | 0.18 | **0.0012** |
| baseline |  | 0.35 | 0.07 | **<.0001** | 0.67 | 0.057 | **<.0001** |
| Age |  | -0.002 | 0.04 | 0.96 | 0.004 | 0.002 | 0.08 |
| Sex | **Female** | -0.91 | 1.25 | 0.47 | 0.01 | 0.069 | 0.87 |
|  | **Male** | 0 |  |  |  |  |  |
| Response | **PAUCI-R** | -0.98 | 1.4 | 0.48 | -0.26 | 0.079 | **0.0024** |
|  | **R+** | 2.41 | 2.11 | 0.26 | -0.44 | 0.119 | **0.0009** |
|  | **R-** | 0 |  |  |  |  |  |
| Time | **1** | 7.26 | 2.06 | **0.0006** | -0.18 | 0.118 | 0.12 |
|  | **2** | 2.33 | 1.9 | 0.22 | -0.053 | 0.108 | 0.62 |
|  | **3** | 2.61 | 1.88 | 0.16 | -0.056 | 0.107 | 0.6 |
|  | **4** | 11.73 | 2.04 | **<.0001** | -0.61 | 0.116 | **<.0001** |
|  | **5** | 0 |  |  |  |  |  |
| IgG |  | -0.0045 | 0.001 | **0.0101** | 0.0002 | 0.0001 | **0.035** |

## Table S7. Associations of Immunoglobulins IgG response with B cells over time

## Multivariable mixed effects modelling revealed associations among IgG and the increase of B cells over time, adjusted for confounders. Estimation for different times. SE: standard error.

| Effect | Response |  |  |  |  |  |  |  |  |  |
| --- | --- | --- | --- | --- | --- | --- | --- | --- | --- | --- |
|  |  | **IgG T1** | | | **IgG T2** | | | **IgG T3** | | |
|  |  | **Estimat.** | **SE** | **P-value** | **Estimat.** | **SE** | **P-value** | **Estimat.** | **SE** | **P-value** |
| Intercept |  | 185.72 | 110.03 | 0.103 | 581.6 | 144.43 | 0.0004 | 239.54 | 137.73 | 0.0923 |
| baseline |  | 18.547 | 6.2344 | 0.0061 | 11.9464 | 3.6843 | 0.0031 | 10.4293 | 3.4088 | 0.0046 |
| Age |  | -3.0457 | 1.5906 | 0.0581 | -3.7251 | 1.9723 | 0.0697 | -1.9383 | 1.8796 | 0.3045 |
| Sex | **Female** | -68.1256 | 48.3842 | 0.1705 | -83.5277 | 70.7483 | 0.248 | -60.9408 | 52.65 | 0.2562 |
|  | **Male** | 0 | . | . | 0 | . | . | 0 | . | . |
| Response | **PAUCI-R** | -2.5869 | 56.8662 | 0.9641 | 18.5818 | 69.6909 | 0.7918 | 1.3136 | 59.3886 | 0.9825 |
|  | **R+** | 315.26 | 99.0822 | 0.0037 | 608.28 | 92.4237 | <.0001 | 618.84 | 85.624 | <.0001 |
|  | **R-** | 0 | . | . | 0 | . | . | 0 | . | . |
| Time | **1** | -495.31 | 72.526 | <.0001 | -592.41 | 89.15 | <.0001 | -554.31 | 78.1354 | <.0001 |
|  | **2** | -278.14 | 73.0315 | 0.0002 | -294.13 | 89.15 | 0.0013 | -269.62 | 78.6803 | 0.0008 |
|  | **3** | -253.47 | 72.526 | 0.0007 | -271.58 | 89.15 | 0.0029 | -261.87 | 78.1354 | 0.0011 |
|  | **4** | -321.2 | 77.4346 | <.0001 | -342.82 | 96.7818 | 0.0006 | -347.36 | 83.6394 | <.0001 |
|  | **5** | 0 | . | . | 0 | . | . | 0 | . | . |
| B cell |  | 36.5419 | 6.8003 | **<.0001** | -2.1793 | 10.4794 | 0.8368 | 33.5287 | 10.017 | **0.0022** |

| Effect | Response |  |  |  |  |  |  |
| --- | --- | --- | --- | --- | --- | --- | --- |
|  |  | **IgG T4** | | | **IgG T5** | | |
|  |  | **Estimat.** | **SE** | **P-value** | **Estimat.** | **SE** | **P-value** |
| Intercept |  | 198.88 | 147.26 | 0.1919 | 426.89 | 125.56 | 0.0023 |
| baseline |  | 20.1336 | 13.9442 | 0.1643 | -20.822 | 12.6375 | 0.1119 |
| Age |  | -2.1681 | 2.1771 | 0.3219 | -3.3558 | 1.5853 | 0.0366 |
| Sex | **Female** | -73.2927 | 60.5762 | 0.2404 | -62.9142 | 49.7164 | 0.2174 |
|  | **Male** | 0 | . | . | 0 | . | . |
| Response | **PAUCI-R** | 69.8263 | 68.3059 | 0.3189 | -32.7006 | 55.1056 | 0.5582 |
|  | **R+** | 480.16 | 129.8 | 0.0014 | 706.57 | 97.2163 | <.0001 |
|  | **R-** | 0 | . | . | 0 | . | . |
| Time | **1** | -516.82 | 85.021 | <.0001 | -496.69 | 68.1602 | <.0001 |
|  | **2** | -284.11 | 85.8675 | 0.0013 | -326.22 | 68.7265 | <.0001 |
|  | **3** | -264.56 | 85.021 | 0.0025 | -300.33 | 68.1602 | <.0001 |
|  | **4** | -336.18 | 84.1686 | 0.0001 | -354.78 | 70.7695 | <.0001 |
|  | **5** | 0 | . | . | 0 | . | . |
| B cell |  | 27.8504 | 8.3314 | **0.0032** | 16.9627 | 8.6214 | 0.0603 |

## 2. Supplementary Figures

**a**

**b**

**Supplementary Figure 1.** **Study design**

**a.** Experimental design and time-point of the study. Study design. Arrows indicate the time-points corresponding to analysis. Blood was collected before immunization (T_0_), and at 24 hours (T_1_), 7 (T_2_), 14 (T_3_), 21 (T_4_) and 28 (T_5_) days after immunization.

**b**. Demographic characteristics: % of sex-matched participants in the longitudinal cohort of CORE study (*N*=46 healthy adult volunteers) in the pre-vaccination stage (T_0_) aged 21–76 years old, mean age 36.05 years old, 3 with a history of SARS-CoV-2 infection) were enrolled and received the BNT162b2 mRNA SARS-CoV-2 vaccine. Chi-squared, P-value (two-tailed) p<0.0001.

**
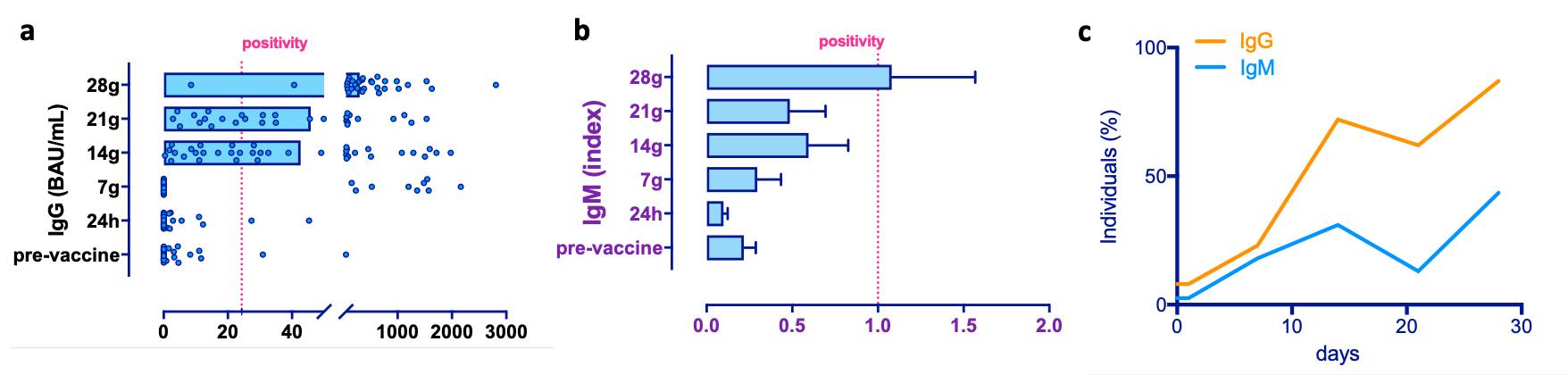
**

**Figure S2. a,** Scatter dot plot of the geometric mean with 95% CI of IgG levels (Binding Antibody Unit/mL), **b,** Bar (one per column) of the geometric mean with 95% CI of IgM levels (Index), during the kinetics of vaccination. Positivity was assessed for IgG values >24.2 (BAU/mL). Reference values IgG: 17.9 – 24.2 doubt, <17.9 negative. **c,** % of increase of the participants with a positive anti-SARS-CoV-2 Immunoglobulins G (orange line), and M (light blue line) tests. The cut-off values to assess positivity is considered under WHO International Standard (20/136) at > 24.2 Binding Antibody Unit (BAU)/mL for IgG, and >= 1 Index for IgM.

**Figure S3. Immunophenotyping for IL4^+^- and IFNg^+^ -producing CD8^+^ T cells subsets and B cells changes in the R^-^, R^+^ and PR stratified groups.**

**
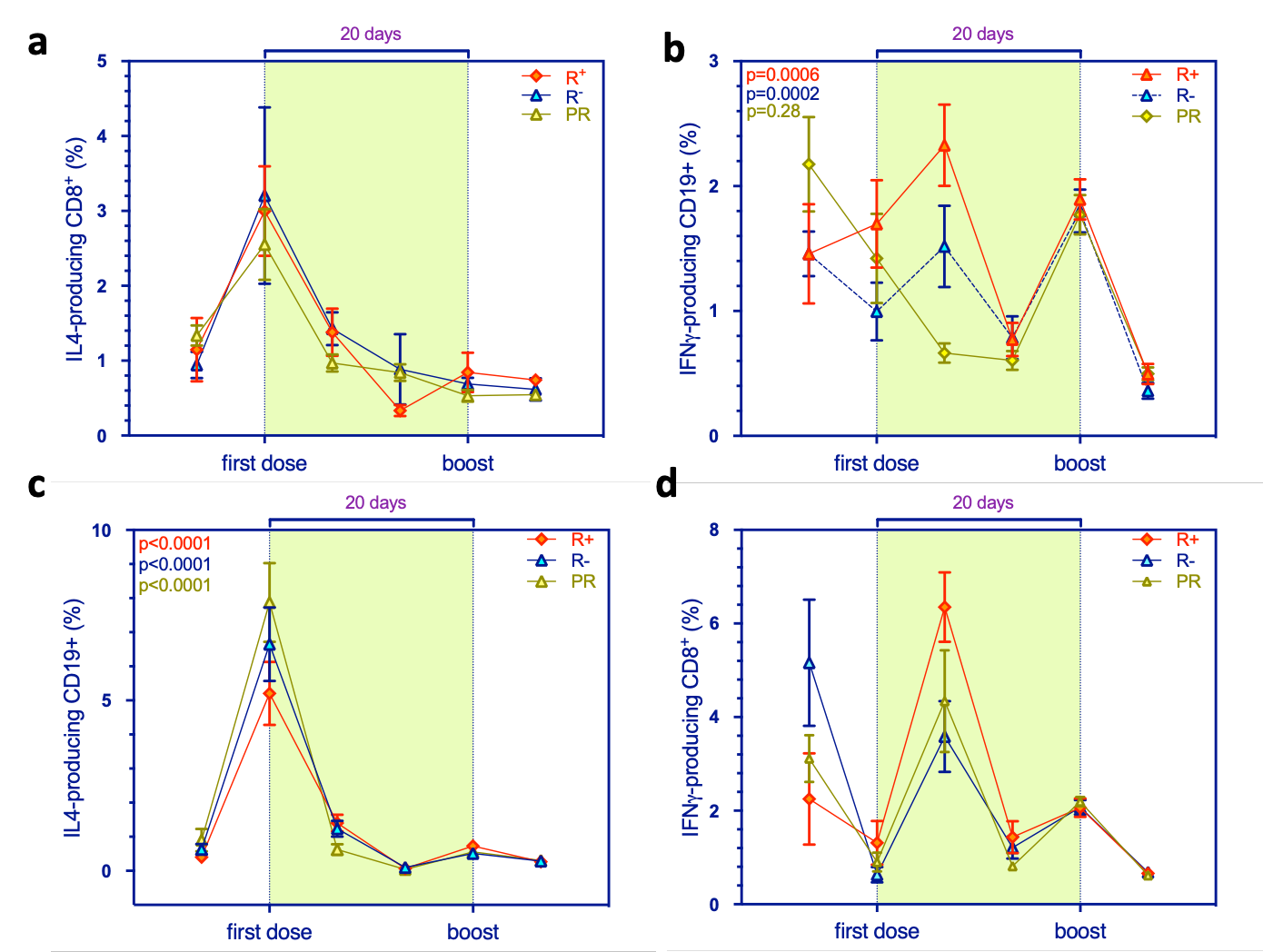
**

**a,** frequency % of IL4^+^ producing CD8^+^**; b,** frequency % of IFNγ^+^ producing B cells (CD19)**; c,** frequency % of IL4^+^ producing B cells (CD19); **d,** frequency % of IFNγ^+^ producing CD8^+^ cells.

**Figure S4. Immunophenotyping for IFNg^+^ and IL4^+^-producing CD4^+^ T cells, and NK subsets changes in the R^-^, R^+^ and PR stratified groups.**

**
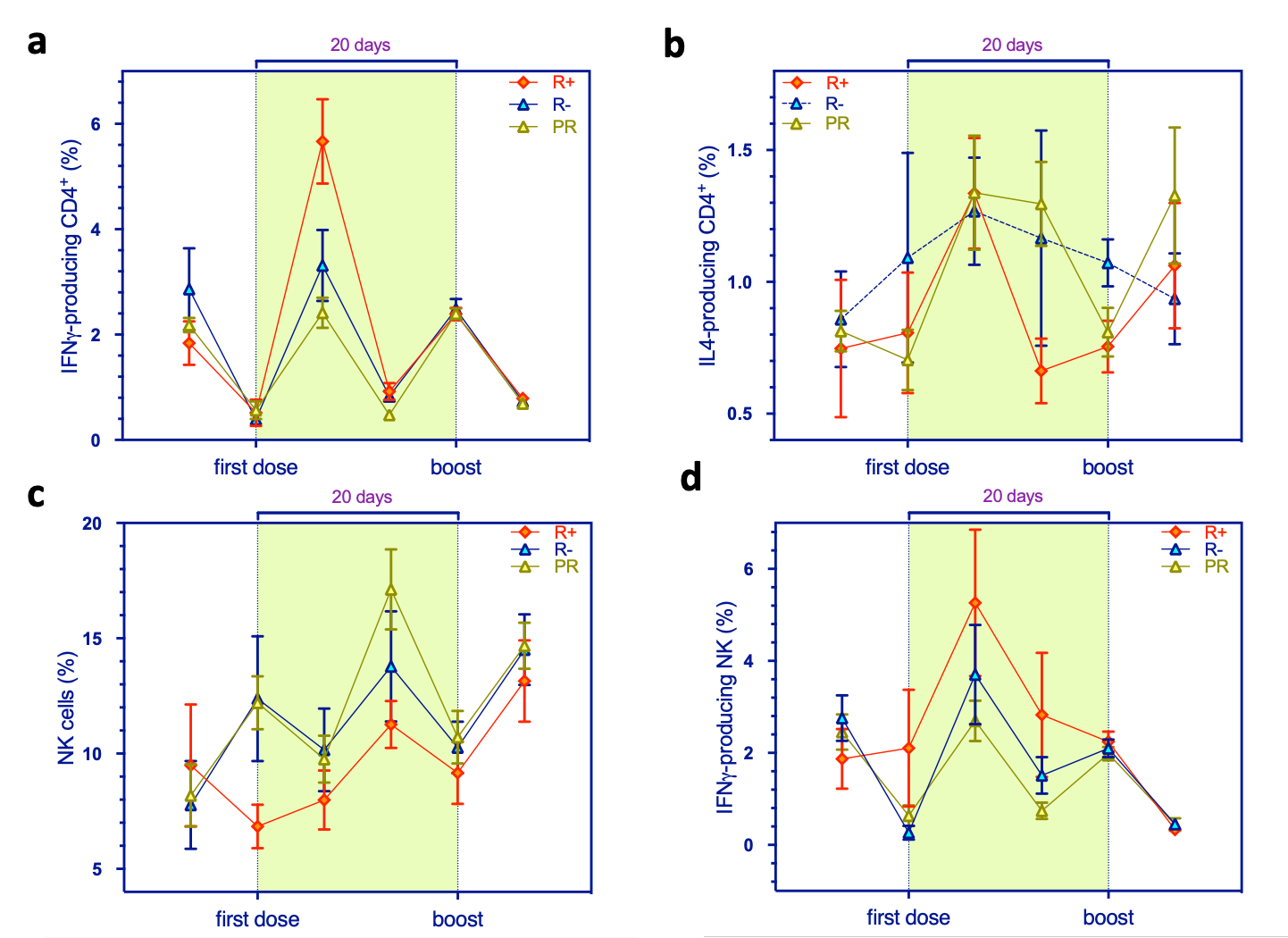
**

**a,** frequency % of IFNγ^+^ producing CD4^+^**; b,** frequency % of IL4^+^ producing CD4^+^ **; c,** frequency % of NK cells; **d,** frequency % of IFNγ^+^ producing NK cells**.**

**Figure S5. Immunophenotyping for B-, T-, and CD4^+^, CD8^+^ T cells subsets changes in the R^-^, R^+^ and PR stratified groups.**

**
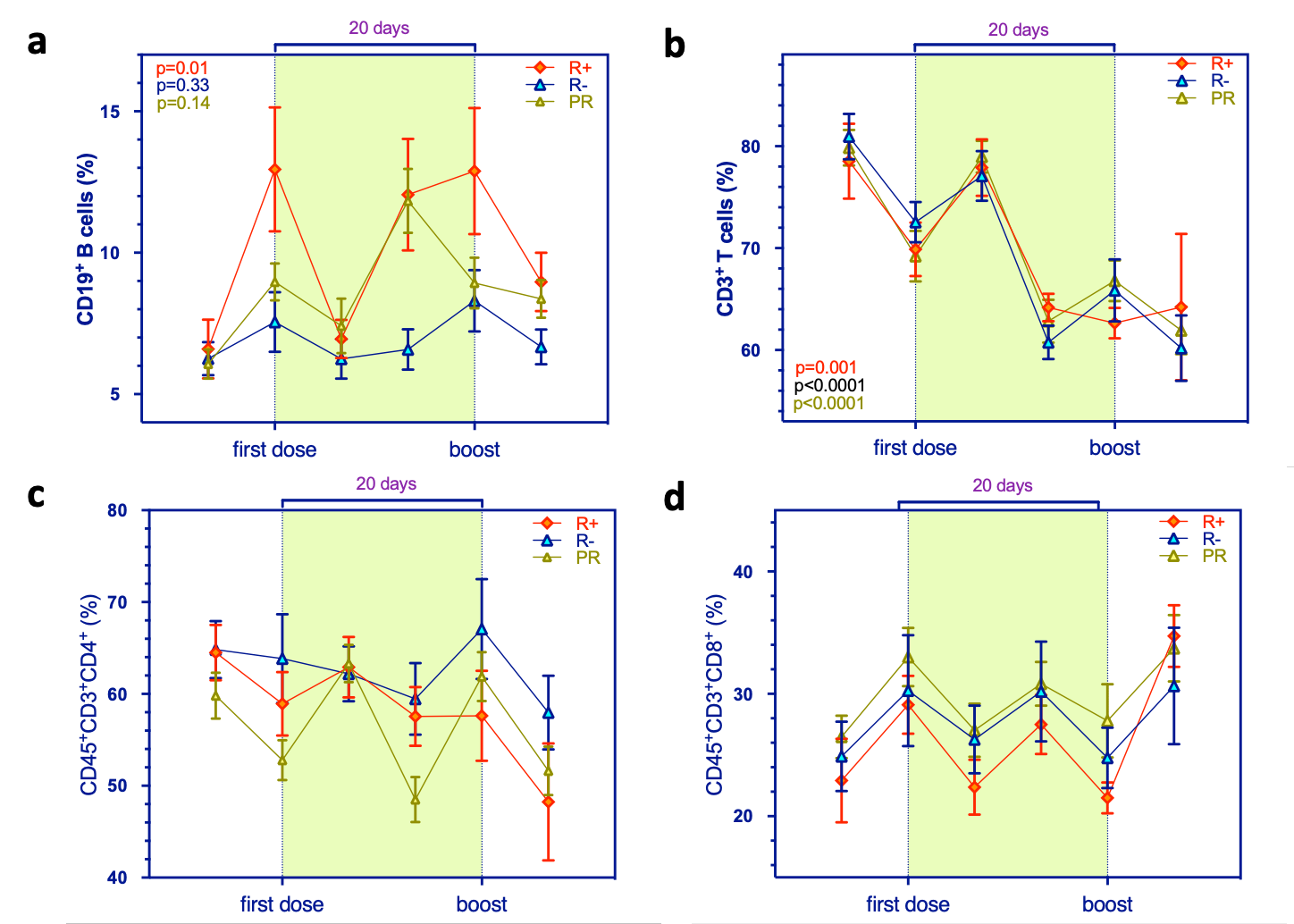
**

**a,** frequency % of B cells (CD19); **b,** frequency % of T cells (CD3); **c,** frequency % of CD4^+^; **d,** frequency % of CD8^+^
