# Supplementary material for: SARS-CoV-2 Immunization Orchestrates the Amplification of IFNγ-Producing T Cell and NK Cell Persistence
Source: Front Immunol. 2022 Feb 14;13:798813. doi: 10.3389/fimmu.2022.798813 (PMC8882867; doi:10.3389/fimmu.2022.798813)
Supplement: Supplementary file 2 [file DataSheet_2.docx]

**Figure S6.** Global pie chart of flow cytometry analysis concerning the frequencies of cytokines-producing immuno-cellular subsets in the time-points vaccination. The % of the selected cell subsets are shown as viable cells (dublets excluded/FSC-A/SSC-A morphologically gated), CD45^+^ total lymphocytes.

**
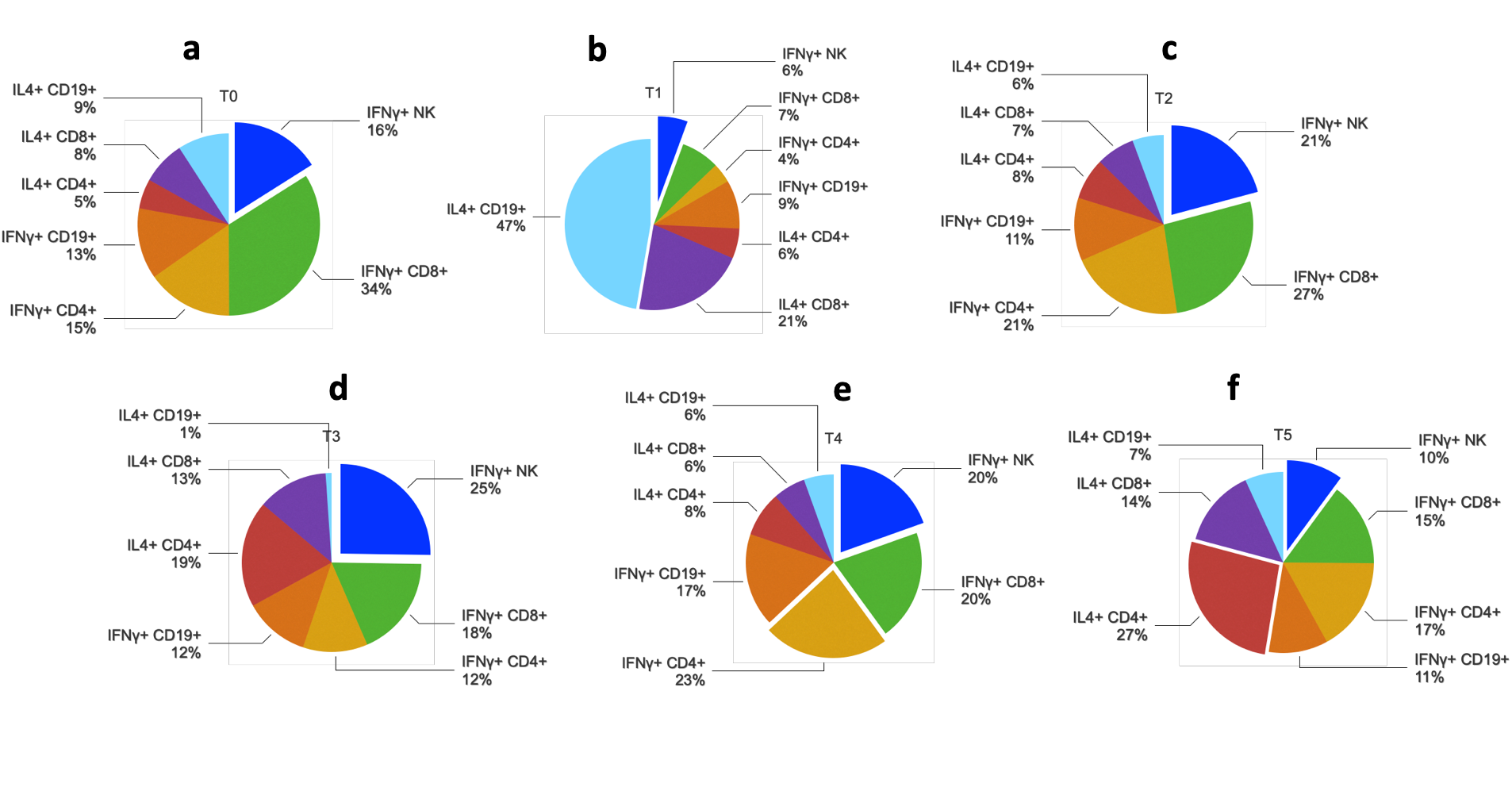
**

**Figure S7. Correlation matrix in different time-points of the vaccine campaign** Heatmaps of the hematological parameters during timing (T_0_-T_5_). Spearman’s rank correlation coefficients are provided for each pair of variables. The correlation plots reported in tables above only significant Spearman coefficient with p<0.01. At different time-points, the IFNγ-producing cells showed changes in correlation analysis, which prevalence is higher in T_1_ with respect to other times.

**
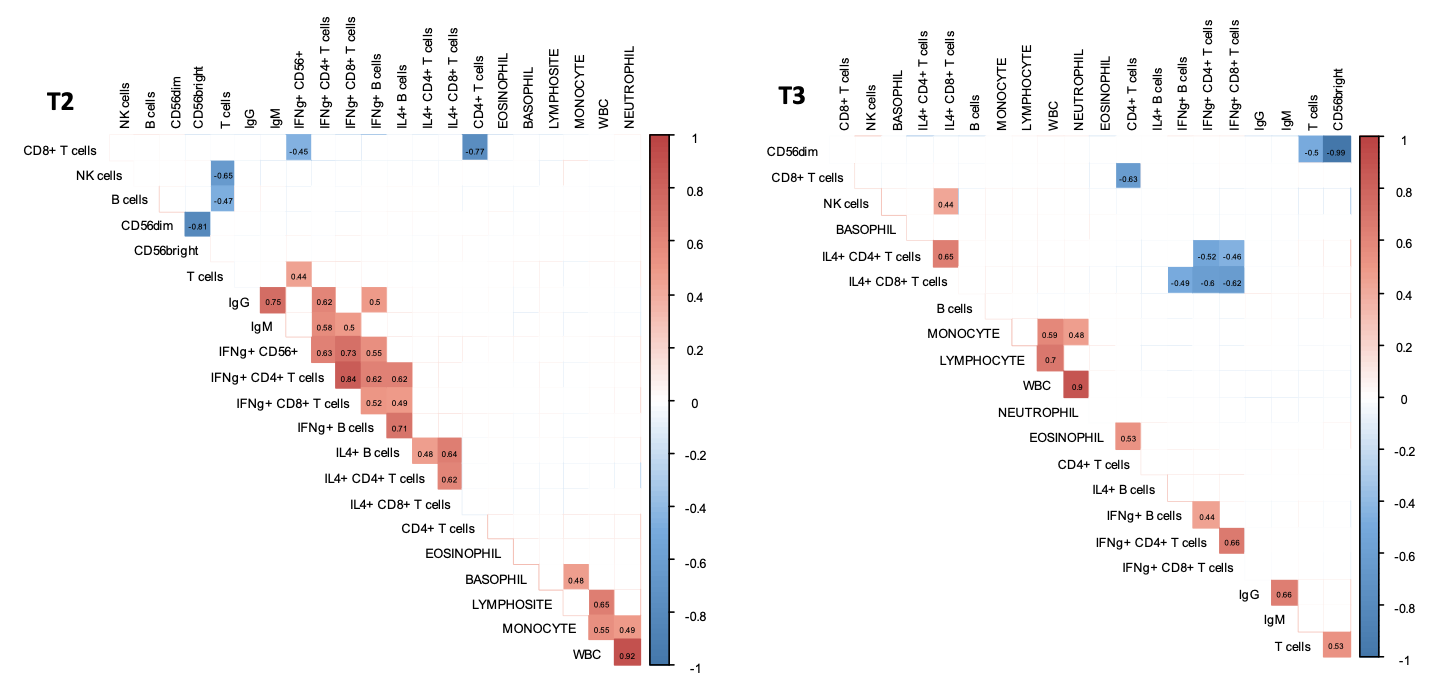
**

**Figure S8. Prevalence of cytokines-producing cells**

Representation of the grading-colour scale of cytokines-producing cells (%) over time.

**Figure S9. Correlation matrix for IgG and CD4, CD8, NK and B cells.** Spearman’s rank correlation coefficients are provided for each pair of variables. The correlation plots reported in tables above only significant Spearman coefficient with p<0.01.

**
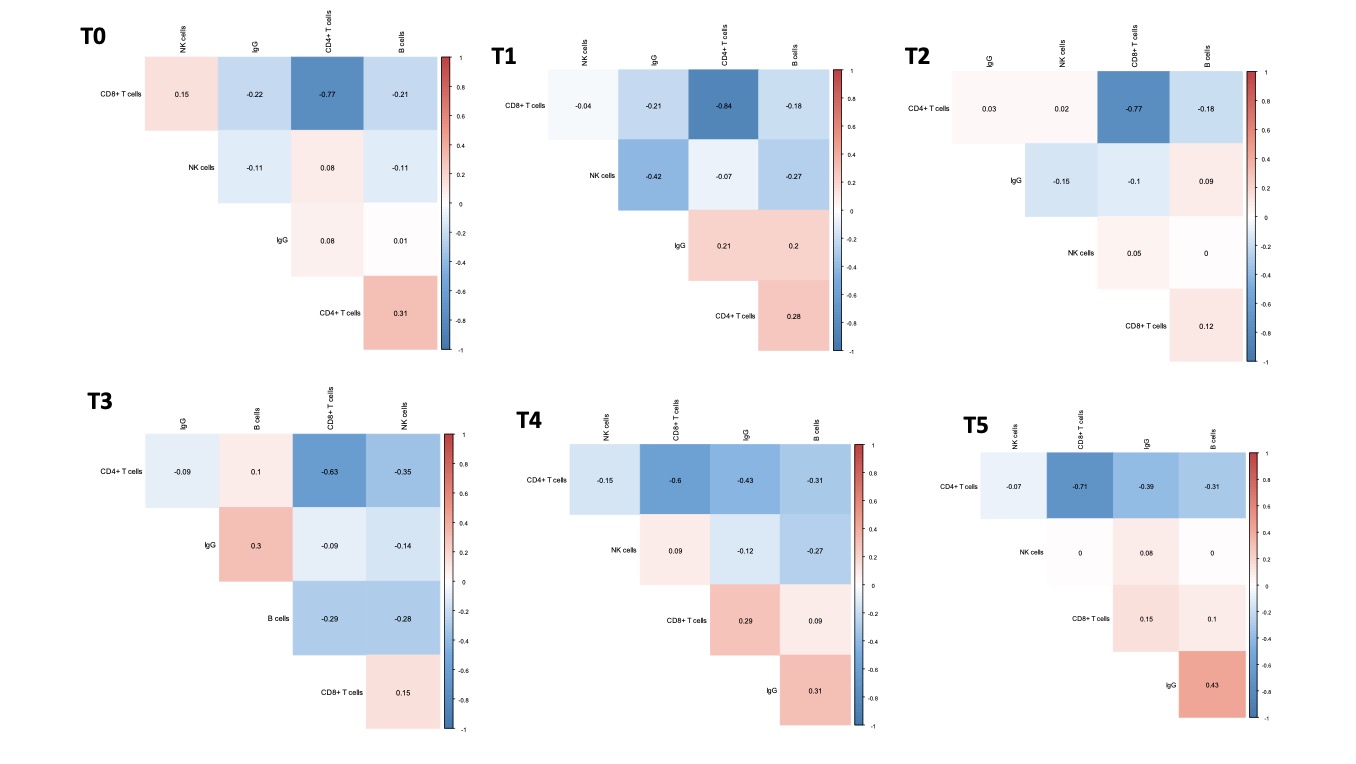
**

**Gating strategy**
